# ONCO-1: Development of a univariate tool based on tumor volume to predict 1-year mortality in patients with oral cavity squamous cell carcinoma

**DOI:** 10.31744/einstein_journal/2025AO1878

**Published:** 2025-11-07

**Authors:** Vitória Marques da Fonseca Morais, Pamela de Oliveira Pinto, Raquel Ajub Moyses, Leonardo Freitas Boaventura Rios

**Affiliations:** 1 Universidade Estadual de Feira de Santana Department of Medicine Feira de Santana BA Brazil Department of Medicine, Universidade Estadual de Feira de Santana, Feira de Santana, BA, Brazil.; 2 Universidade de São Paulo Faculdade de Medicina Laboratório de Cirurgia de Cabeça e Pescoço São Paulo SP Brazil Laboratório de Cirurgia de Cabeça e Pescoço - LIM28, Faculdade de Medicina, Universidade de São Paulo, São Paulo, SP, Brazil.

**Keywords:** Mouth neoplasms, Carcinoma, squamous cell, Tumor burden, Prognosis, Mortality, Logistic models, Decision trees

## Abstract

**Objective:**

To develop a simple and accessible univariate tool based on tumor volume to predict 1-year mortality in patients with oral cavity squamous cell carcinoma.

**Methods:**

A retrospective analysis was conducted on patients with oral cavity squamous cell carcinoma included in the GENCAPO cohort. Tumor volume was calculated using the formula of Monga et al. Classification and regression trees were used to define risk categories after determining the volume cutoff points. Logistic regression using 1-year vital status (alive or deceased) as the outcome was employed to construct the Oral Neoplasm Clinical Outcome in 1 year (ONCO)-1 score. Model discrimination and calibration were evaluated using the area under the receiver operating characteristic curve and the Hosmer-Lemeshow test, respectively. After building the model, we compared it with the TNM system (8th edition) using Kaplan-Meier curves.

**Results:**

ONCO-1 showed good stratification ability, with a progressive increase in odds ratios for 1-year mortality: odds ratio 2.47 (G2), 4.01 (G3), and 9.99 (G4), compared to group G1. The model demonstrated good discrimination (area under the curve=0.7031) and excellent calibration (Hosmer-Lemeshow p=0.99978). Kaplan-Meier curves indicated a superior prognostic performance of ONCO-1 compared to TNM classification in predicting 1-year survival.

**Conclusion:**

Tumor volume proved to be a robust predictor of short-term mortality in oral cavity squamous cell carcinoma. ONCO-1 provided better 1-year risk stratification compared to TNM and may contribute to more individualized clinical decision-making.

## In Brief

A univariate logistic model based on tumor volume, ONCO-1, was capable of predicting the 1-year survival of patients with oral cavity squamous cell carcinoma. Its prognostic performance was superior to the current TNM model, demonstrating better predictive ability compared to a 12-month adaptation of TNM. This suggests that tumor volume should be considered as a prognostic factor and may be useful in clinical decision-making.

## Highlights

■ We developed ONCO-1, a simple tool based on tumor volume.
■ Cutoff points were defined by a CART model.
■ Tumor volume was a prognostic factor for 1-year mortality.
■ Tumor volume can to support clinical decision-making.

## Graphical abstract

**Figure:**
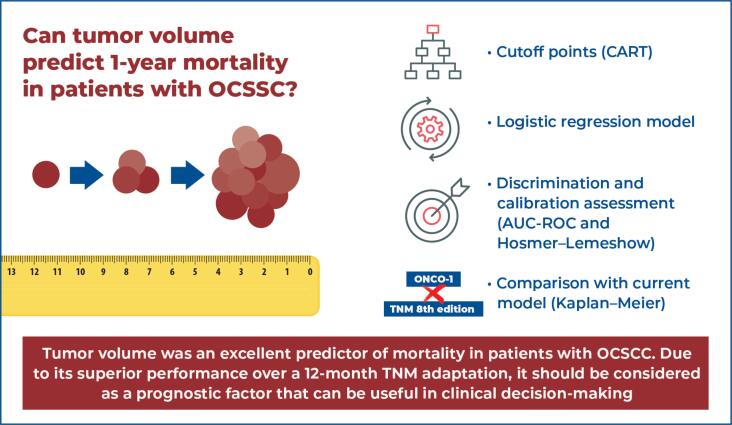

## INTRODUCTION

Head and neck cancer is currently the sixth most common type of cancer worldwide.^(1)^ According to GLOBOCAN 2022, when combining data from cancers of the oral cavity, larynx, oropharynx, and hypopharynx, 771,694 new cases were reported.^(2)^ In Brazil, the annual estimate for the 2023-2025 period from the National Cancer Institute (INCA - *Instituto Nacional de Câncer*) was 15,100 new cases of oral cavity carcinoma (OCC), corresponding to an estimated risk of 6.99 cases per 100,000 inhabitants.^(3)^ Regarding histological type, 90% of diagnoses are squamous cell carcinoma (SCC).^(4)^

Greater accuracy in the clinical staging of oral cavity squamous cell carcinoma (OCSCC) is a priority to optimize prognostic evaluation and ensure that patients receive appropriate care. For OCSCC, the staging system used is the TNM classification of the American Joint Committee on Cancer, 8th edition. This system considers the anatomical aspects of the primary tumor (T), regional lymph node metastases (N), and distant metastases (M), subsequently classifying the patient into four main stages.^(5)^ However, because the classification is based on risk categories, the stage assigned to a given patient does not always correspond to an accurate estimate of an individual's risk of death. Thus, one of the main criticisms of the TNM system is the grouping of heterogeneous samples of patients, in which individuals with distinct prognoses are allocated to the same group.^(6)^

Accurate short-term prognostic evaluation allows for better access to and mobilization of resources, and contributes to the development of health policy changes.^(7)^ However, the TNM system only estimates 5-year survival, offering no data on shorter-term outcomes. It is important to consider that patients and families should actively participate in therapeutic decisions once knowledge of short-term prognosis enables a clearer understanding of disease progression, allowing for more informed choices that are compatible with patient's personal values and care priorities.^(8)^

In this context, tumor volume has been investigated as an important predictive factor for appropriate staging of OCSCC, showing significant value in treatment outcome evaluation, and serving as an independent factor for disease-free and overall survival.^(9,10)^ Some studies suggest that tumor volume calculated through imaging methods may be a better predictor of overall survival than the TNM system.^(11,12)^

The choice of imaging method for proper evaluation of OCSCC should consider not only diagnostic precision, but also economic feasibility and method availability. Contrast-enhanced computed tomography (CT) is capable of assessing tumor invasion, bone involvement, and cervical lymph node metastases.^(13)^ Magnetic resonance imaging (MRI) has superior soft tissue assessment capacity and may be more effective in detecting perineural invasion; however, it is less accessible.^(14)^ Positron emission tomography (PET/CT), despite its high sensitivity for detecting cervical nodal metastases, presents difficulties in terms of availability and financial viability. Thus, contrast-enhanced CT has become the most practical and accessible choice for evaluating OCSCC.^(15)^

Accurate clinical staging of OCSCC is essential to ensure a more precise prognostic evaluation and, consequently, better patient care. In this context, a key question arises: is it possible to develop a simple, univariate tool based on tumor volume capable of predicting 1-year mortality in patients with OCSCC?

## OBJECTIVE

To develop ONCO-1 (Oral Neoplasm Clinical Outcome in 1 year), a predictive model based solely on tumor volume.

## METHODS

This was a retrospective cohort study including patients prospectively enrolled in the database of the Head and Neck Cancer Genome Project (GENCAPO - *Genoma do Câncer de Cabeça e Pescoço*) between July 4, 2000, and August 16, 2011, diagnosed with OCSCC. We analyzed the data of patients diagnosed with OCSCC in four hospital centers in the state of São Paulo, Brazil.

The data used are part of a subproject of the study "Environmental, clinical, histopathological and molecular factors associated with the development and prognosis of head and neck squamous carcinomas", conducted in the Department of Surgery of the *Hospital das Clínicas*, *Faculdade de Medicina, Universidade de São Paulo* (FMUSP), and previously approved by the Ethics Committee for Analysis of Research Projects (CAPPesq - *Comissão de Ética para Análise de Projetos de Pesquisa*) under No. 0511/07. The study was conducted in accordance with the ethical standards of the institutional and national research committee. All participants provided written informed consent at the time of inclusion.

Data on the three tumor dimensions (length, width, and depth) were obtained at the time of diagnosis. After data collection, some patients were identified as having missing information regarding tumor dimensions. To avoid excluding these patients from the study, data imputation was performed using shrinkage and regression techniques, preserving the variability of the dataset. Initially, a linear regression model was applied using pathological anatomy measurements from patients with available data. When this was not feasible, the mean of available measurements in each clinical staging category and tumor site was used. In cases where none of the methods were able to impute the clinical dimensions, we applied a shrinkage covariance estimator. Tumor volume was subsequently analyzed using the formula described by Monga et al.^(16)^ [(length (DC1) × width (DC2) × depth (thickness) /2)]. The tumor volume measurement and its predictive value for 1-year patient outcomes were considered.

A Classification and Regression Trees (CART) model was used to categorize tumor volume, defining three cutoff points and four risk categories. A logistic regression model was then constructed using the four groups to create a predictive tool. To evaluate the accuracy of this model, the area under the receiver operating characteristic curve (AUC) was used for discrimination,^(17)^ and the Hosmer-Lemeshow test was applied for calibration.^(18)^ Finally, the outcomes predictive capacity of ONCO-1 was compared to that of the TNM 8th edition using Kaplan-Meier survival curves.^(19)^

All statistical analyses were performed using JMP Pro 10.0.0 (SAS Institute Inc., Cary, NC, USA).

## RESULTS

The study involved 752 patients diagnosed with OCSCC between July 4, 2000, and August 16, 2011. The patients were mostly male, in the fifth and sixth decades of life, with a history of smoking and alcohol use, as described in table 1.

**Table 1:** Demographic data of the patients with oral cavity squamous cell carcinoma included in the study (n=752)

Variable		n (%)
Sex		
	Female	144 (19.10)
	Male	607 (80.80)
	Unknown	1
Age (years)		
Mean [range]		57 [21-93]
Smoking history		660 (88.80)
Alcohol use		615 (83.00)

**Figure 1:**
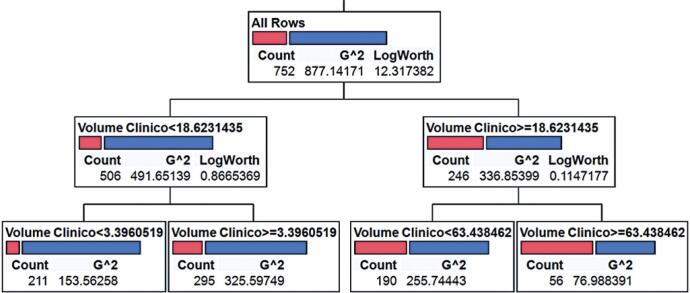
Classification and Regression Trees (CART) model based on tumor volume to define risk categories for 1-year mortality

The mean tumor volume of the patients was 23.45cm^3^±1.66cm^3^. Using a CART model, the initial cutoff point identified was 18.62cm^3^, and after secondary divisions, three final cutoff points were established: 3.4cm^3^, 18.62cm^3^, and 63.43cm^3^, resulting in four risk categories based on mortality (Figure 1).

Cutoff points determined: 3.0 cm^3^, 18.0 cm^3^, and 60.0 cm^3^, creating four ONCO-1 risk groups.

**Figure 2:**
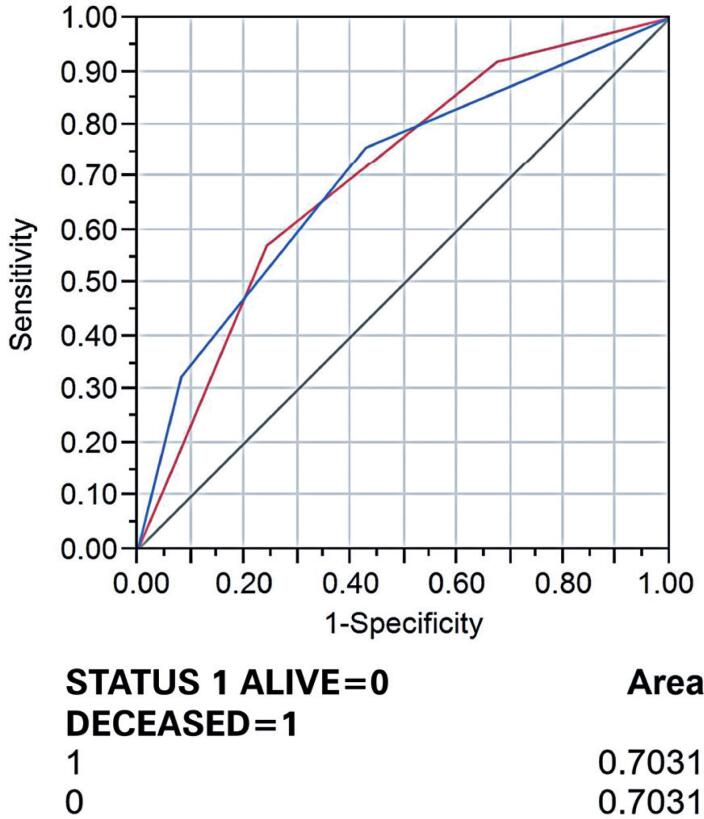
Receiver operating characteristic curve of the ONCO-1 logistic regression model

The groups were designated as: G1 ≤3.0cm^3^; 3.0cm^3^<G2 ≤18.0cm^3^; 18.0cm^3^<G3 ≤60cm^3^; and G4 >60cm^3^ (Table 2). Following logistic regression analysis, odds ratios were determined (Table 3). Once the cutoff points were defined, the area under the curve (AUC) was used to test the model's discrimination ability. An AUC value of 0.7031 was determined (Figure 2).

**Table 2:** Distribution of patients across the tumor volume-based risk categories

Tumor volume	
Mean	23.45cm^3^
G1 (≤ 3.0 cm^3^), n (%)	199 (26.40)
G2 (3.0-18.0 cm^3^), n (%)	306 (40.70)
G3 (18.0-60 cm^3^), n (%)	189 (25.10)
G4 (> 60 cm^3^), n (%)	57 (7.50)

Distribution of patients across tumor volume-based risk categories derived from Classification and Regression Trees (CART) analysis. Cutoff points: G1 ≤3.0cm^3^, G2=3.0-18.0cm^3^, G3=18.0-60.0cm^3^, and G4 >60.0 cm^3^.

**Table 3:** Logistic regression analysis

Risk category	Odds ratio	95% CI	pvalue
G1	-	-	
G2	2.47	1.29-4.96	0.0054
G3	4.01	2.02-8.34	<0.0001
G4	9.99	3.72-28.4	<0.0001

Results of univariate logistic regression model predicting 1-year mortality according to ONCO-1 tumor volume risk groups. G1 was used as a reference. Odds ratios (OR) with 95% confidence intervals (CI) and p-values are shown.

The area under the curve was 0.7031, demonstrating good discriminative ability for 1-year mortality prediction.

**Figure 3:**
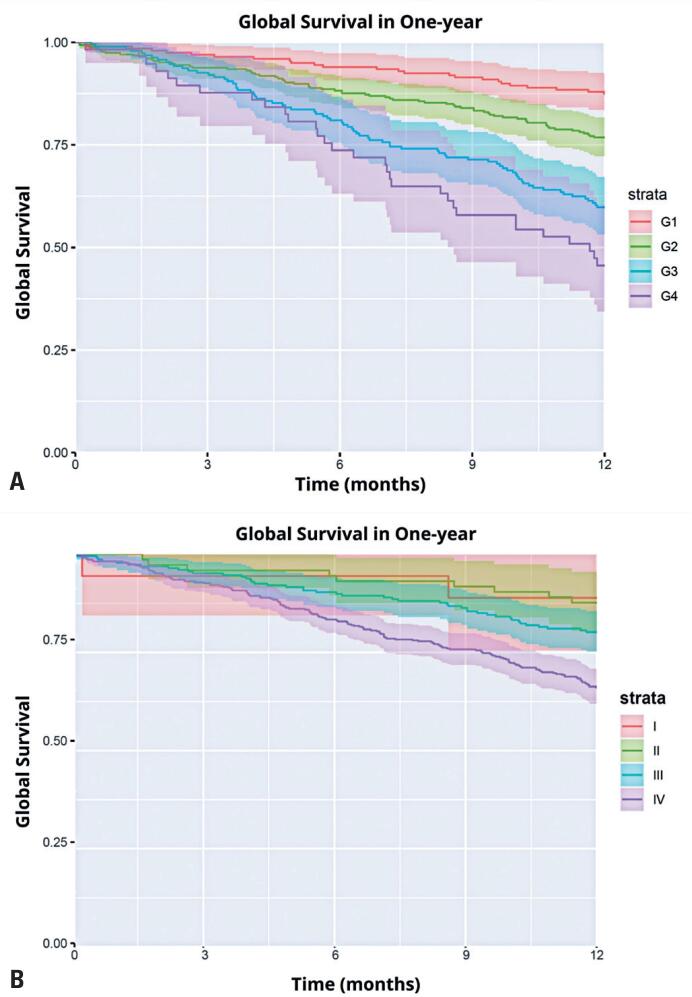
Kaplan-Meier survival curves comparing ONCO-1 (A) and TNM (8th edition) (B) systems in predicting 1-year survival in OCSCC compared to the TNM

The predictive performance of the model in terms of calibration was assessed using the Hosmer-Lemeshow test, with a p-value of 0.99978 (Table 4). Kaplan-Meier survival curves comparing ONCO-1 and TNM revealed that ONCO-1 had greater accuracy in outcome prediction for 1-year survival outcomes compared to TNM (Figure 3).

**Table 4:** Hosmer-Lemeshow test for calibration of the logistic regression model

Calibration	Risk category	Mean risk	Observed mortality
0-25%	G1	0.15	0.13
25.1-50%	G2	0.26	0.23
50.1-75%	G3	0.41	0.40
75.1-100%	G4	0.58	0.54
p value			0.99978

Calibration of the logistic regression model by ONCO-1 risk category using the Hosmer-Lemeshow test. Comparison between mean predicted risk and observed 1-year mortality. Excellent model calibration was indicated by a p-value of 0.99978.

ONCO-1 shows improved stratification capacity and better prognostic performance compared to the TNM.

## DISCUSSION

In this study we demonstrate that the univariate logistic model based on tumor volume, ONCO-1, was capable of predicting the 1-year survival of patients with OCSCC. ONCO-1 was superior to the currently used TNM model, demonstrating better predictive ability compared to a 12-month adaptation of TNM.

Treatment of OCSCC varies according to stage and severity, with final decisions made by a multidisciplinary team considering tumor control, functionality, and patient quality of life. Surgery is the treatment of choice for all resectable cases, including locally advanced tumors. In early-stage cases, surgery alone is usually sufficient, with no need for additional therapies. In locally advanced disease, the approach becomes more complex and treatment is generally multimodal, with the possibility of combining radiotherapy or chemotherapy to improve local control and survival.^(20,21)^ In cases of very advanced or unresectable disease, chemoradiotherapy may be used as an attempt at locoregional control, although this strategy has limited success rates and high toxicity.^(22)^

Thus, OCSCC treatment presents points of discussion, particularly concerning the balance between disease control and functionality preservation.^(23,24)^ One example is the extent and necessity of adjuvant treatment in patients with intermediate-risk features, who may not benefit from aggressive additional therapies.^(25)^ Another issue regarding treatment adequacy is the TNM grouping system into broad risk categories, where individuals with different profiles may share the same stage, especially in locally advanced cases.^(26)^ This hinders therapeutic decisions because these patients may follow different prognoses depending on their individual characteristics.^(27,28)^ The need for precise prognostic information and therapeutic planning encourages the search for tools that enhance staging accuracy.

Previous studies have raised the possibility of using tumor volume as a tool for prognostic assessment. While some authors, such as Tofanelli et al., suggest that tumor volume may be used as a complement to the TNM system,^(29)^ others indicate that it functions as an independent risk factor for prognostic evaluation, showing greater accuracy in predicting the risk of nodal metastases and extracapsular spread than the TNM in OCSCC staging.^(30)^

Use of tumor volume as a prognostic marker has also been studied in other cancer sites. In gastric cancer, it outperformed tumor diameter and T-stage, improving prognostic precision.^(31)^ In non-small cell lung cancer, tumor volume was a better predictor of overall survival than tumor diameter.^(32)^ In colorectal cancer, CT-based volume assessment correlated significantly with disease stage and patient survival.^(33)^

Staging accuracy in OCSCC influences both prognosis and treatment planning. Detailed assessment of tumor extension is crucial for surgical planning,^(34,35)^ and accurate staging avoids unnecessary treatments and associated morbidity.^(36)^ CT-based volume measurement supports practical and reproducible application, especially in low-resource settings where MRI and PET/CT are less accessible.^(37)^ CT also offers good anatomical definition compared to MRI and PET/CT. In oral cavity tumors, the puffed-cheek technique–asking patients to inflate their cheeks during imaging–enhances delineation, especially for tumors along the gingiva and buccal mucosa.^(38)^

In this study, a decision tree model (CART) was applied a retrospective cohort of patients diagnosed with OCSCC to define three cutoff points for tumor volume, resulting in four risk categories for 1-year mortality. Patients were allocated according to tumor volume, and when comparing the odds ratios among the groups, a clear trend of progressively increased mortality risk was observed as tumor volume increased. Patients classified in the highest risk group (G4) had a tenfold greater probability of death within 1 year compared to those in the lowest risk group (G1), reinforcing the prognostic relevance of volume as a continuous variable. Likewise, intermediate groups (G3 and G2), when compared to G1, showed fourfold and twofold higher chances, respectively, of adverse outcomes within the evaluated period. These findings highlight the ability of the ONCO-1 to adequately stratify patients based on a single, objective, and accessible parameter, reflecting a consistent risk gradient. Moreover, they suggest that tumor volume is a valuable marker to guide individualized clinical decisions by capturing nuances in disease extent that the TNM system may not discriminate well.

Kaplan-Meier curves showed that ONCO-1 more accurately predicted 1-year outcomes than the 12-month adapted TNM system. Survival analysis demonstrated that patients classified by ONCO-1 had significantly different survival rates over the 12-month period, showing superior risk discrimination. This finding enables ONCO-1 to overcome the limitations of TNM in providing short-term predictions.

This study offers valuable clinical contributions due to the high quality of prospectively collected data by trained interviewers and the practicality of the tool developed. ONCO-1 uses tumor volume, a simple, accessible, and replicable measure. This allows more objective and standardized prognostic assessment, providing physicians and researchers with a reliable resource in the prognostic evaluation of OCSCC.

However, the study has a few limitations. We are unaware of a specific 1-year prognostic model that could serve as a direct reference, which makes accurate comparison difficult. Furthermore, the TNM system, used here in a 1-year simulation, was originally designed to evaluate 5-year outcomes, which may have influenced the comparison results with ONCO-1.

## CONCLUSION

Regarding the central question of whether tumor volume can independently predict 1-year mortality in patients with oral cavity squamous cell carcinoma, we found that tumor volume is indeed a robust prognostic factor. Based on this variable as a foundation and employing machine learning techniques combined with logistic regression, it was possible to develop a univariate logistic model. ONCO-1 showed good discrimination and calibration for predicting 1-year survival, based on easily retrievable information, which outperformed the TNM system. These findings suggest that tumor volume should be considered a prognostic factor and may be useful in clinical decision-making. External validation of these findings is required to ensure that ONCO-1 can be consistently applied in different settings. Moreover, these findings should be compared to other prognostic models specifically designed for 12-month outcomes, aiming to provide a reliable and accessible tool to support effective and individualized patient risk stratification and care.

## References

[1] Johnson DE, Burtness B, Leemans CR, Lui VW, Bauman JE, Grandis JR (2020). Head and neck squamous cell carcinoma. Nat Rev Dis Primers.

[2] Bray F, Laversanne M, Sung H, Ferlay J, Siegel RL, Soerjomataram I (2024). Global cancer statistics 2022: GLOBOCAN estimates of incidence and mortality worldwide for 36 cancers in 185 countries. CA Cancer J Clin.

[3] Instituto Nacional de Câncer (INCA) (2022). Estimativa 2023: Incidência de câncer no Brasil.

[4] D’Silva NJ, Gutkind JS (2019). Oral cancer: integration of studies for diagnostic and therapeutic precision. Adv Dent Res.

[5] Almangush A, Mäkitie AA, Triantafyllou A, de Bree R, Strojan P, Rinaldo A (2020). Staging and grading of oral squamous cell carcinoma: an update. Oral Oncol.

[6] Wolfensberger M (1992). Using Cox's proportional hazards model for prognostication in carcinoma of the upper aero-digestive tract. Acta Otolaryngol.

[7] Hum A, Wong YK, Yee CM, Lee CS, Wu HY, Koh MY (2020). PROgnostic model for advanced cancer (PRO-MAC). BMJ Support Palliat Care.

[8] Tanaka OY, Tamaki EM (2012). O papel da avaliação para a tomada de decisão na gestão de serviços de saúde. Cien Saude Colet.

[9] Pameijer FA, Balm AJ, Hilgers FJ, Muller SH (1997). Variability of tumor volumes in T3-staged head and neck tumors. Head Neck.

[10] Mijatov I, Kiralj A, Ilić MP, Vučković N, Spasić A, Nikolić J (2023). Pathological tumor volume as a simple quantitative predictive factor of survival in oral squamous cell carcinoma. Oncol Lett.

[11] Mair M, Nair D, Nair S, Malik A, Mishra A, Kannan S (2018). Comparison of tumor volume, thickness, and T classification as predictors of outcomes in surgically treated squamous cell carcinoma of the oral tongue. Head Neck.

[12] Mukherji SK, O’Brien SM, Gerstle RJ, Weissler M, Shockley W, Stone JA (2000). The ability of tumor volume to predict local control in surgically treated squamous cell carcinoma of the supraglottic larynx. Head Neck.

[13] Cervenka B, Pipkorn P, Fagan J, Zafereo M, Aswani J, Macharia C (2019). Oral cavity cancer management guidelines for low-resource regions. Head Neck.

[14] Arya S, Rane P, Deshmukh A (2014). Oral cavity squamous cell carcinoma: role of pretreatment imaging and its influence on management. Clin Radiol.

[15] Ng SH, Yen TC, Liao CT, Chang JT, Chan SC, Ko SF (2005). 18F-FDG PET and CT/MRI in oral cavity squamous cell carcinoma: a prospective study of 124 patients with histologic correlation. J Nucl Med.

[16] Monga SP, Wadleigh R, Sharma A, Adib H, Strader D, Singh G (2000). Intratumoral therapy of cisplatin/epinephrine injectable gel for palliation in patients with obstructive esophageal cancer. Am J Clin Oncol.

[17] DeLong ER, DeLong DM, Clarke-Pearson DL (1988). Comparing the areas under two or more correlated receiver operating characteristic curves: a nonparametric approach. Biometrics.

[18] Hosmer DW, Hosmer T, Le Cessie S, Lemeshow S (1997). A comparison of goodness-of-fit tests for the logistic regression model. Stat Med.

[19] Rich JT, Neely JG, Paniello RC, Voelker CC, Nussenbaum B, Wang EW (2010). A practical guide to understanding Kaplan-Meier curves. Otolaryngol Head Neck Surg.

[20] Metzger K, Moratin J, Horn D, Pilz M, Ristow O, Hoffmann J (2021). Treatment delay in early-stage oral squamous cell carcinoma and its relation to survival. J Craniomaxillofac Surg.

[21] Silva LC, Faustino IS, Ramos JC, Colafemina AC, Di Pauli-Paglioni M, Leite AA (2023). The importance of early treatment of oral squamous cell carcinoma: case report. Oral Oncol.

[22] Ghorbanpour M, Salarvand S, Salarvand S, Shahsavari F, Shirkhoda M, Shakib PA (2024). Depth of invasion and extranodal extension: the influential factors to predict survival rate of patients with oral tongue squamous cell carcinoma. BMC Cancer.

[23] Julian RS, Woo BM, Rabey EC (2021). Oral cavity and oropharyngeal cancer: treatment. J Calif Dent Assoc.

[24] Asarkar AA, Chang BA, de Bree R, Kowalski LP, Guntinas-Lichius O, Bradley PJ (2024). Primary management of operable locally advanced oral cavity squamous cell carcinoma: current concepts and strategies. Adv Ther.

[25] Yamada SI, Kurita H, Nakano R, Ohta R, Akita D, Hashidume M (2020). Treatment strategies for and outcomes of older patients with oral squamous cell carcinoma. Oral Surg Oral Med Oral Pathol Oral Radiol.

[26] Patel EJ, Oliver JR, Vaezi A, Li Z, Persky M, Tam M (2021). Primary surgical treatment in very advanced (T4b) oral cavity squamous cell carcinomas. Otolaryngol Head Neck Surg.

[27] Matos LL, Guimarães YL, Leite AK, Cernea CR (2023). Management of stage III oral cavity squamous cell carcinoma in light of the new staging system: a critical review. Curr Oncol Rep.

[28] de Almeida JR, Su JS, Kolarski MM, Truong T, Weinreb I, Perez-Ordonez B (2024). Development and validation of a novel TNM staging N-classification of oral cavity squamous cell carcinoma. Cancer.

[29] Tofanelli M, Boscolo Nata F, Giudici F, Cadenar A, Gardenal N, Marcuzzo AV (2023). Is there a role for tumor volume in prediction of prognosis for oral cancer?. Am J Otolaryngol.

[30] Kjems J, Elisabet Håkansson K, Andrup Kristensen C, Grau Eriksen J, Horsholt Kristensen M, Ivalu Sander Holm A (2023). The influence of tumor volume on the risk of distant metastases in head and neck squamous cell carcinomas. Radiother Oncol.

[31] Liu Z, Gao P, Liu S, Zheng G, Yang J, Sun L (2017). Tumor volume increases the predictive accuracy of prognosis for gastric cancer: A retrospective cohort study of 3409 patients. Oncotarget.

[32] Xie HJ, Zhang X, Mo YX, Long H, Rong TH, Su XD (2019). Tumor volume is better than diameter for predicting the prognosis of patients with early-stage non-small cell lung cancer. Ann Surg Oncol.

[33] Park JY, Kim SH, Lee SM, Lee JS, Han JK (2017). CT volumetric measurement of colorectal cancer helps predict tumor staging and prognosis. PLoS One.

[34] Lee NC, Eskander A, Park HS, Mehra S, Burtness BA, Husain Z (2019). Pathologic staging changes in oral cavity squamous cell carcinoma: stage migration and implications for adjuvant treatment. Cancer.

[35] Lam V, O’Brien O, Amin O, Nigar E, Kumar M, Lingam RK (2024). Oral cavity cancer and its pre-treatment radiological evaluation: A pictorial overview. Eur J Radiol.

[36] Linz C, Brands RC, Herterich T, Hartmann S, Müller-Richter U, Kübler AC (2021). Accuracy of 18-F fluorodeoxyglucose positron emission tomographic/computed tomographic imaging in primary staging of squamous cell carcinoma of the oral cavity. JAMA Netw Open.

[37] Guenette JP (2021). Radiologic evaluation of the head and neck cancer patient. Hematol Oncol Clin North Am.

[38] Gule-Monroe MK, Calle S, Policeni B, Juliano AF, Agarwal M, Chow LQM, Dubey P, Friedman ER, Hagiwara M, Hanrahan KD, Jain V, Rath TJ, Smith RB, Subramaniam RM, Taheri MR, Yom SS, Zander D, Burns J, Expert Panel on Neurological Imaging (2023). ACR Appropriateness Criteria® Staging and Post-Therapy Assessment of Head and Neck Cancer. J Am Coll Radiol.

